# Progress in State Medicine

**Published:** 1901-12-28

**Authors:** 


### Progress in State Medicine.

### HOUSING OF THE WORKING CLASSES.

Chisholm,[1] speaking at the Glasgow Conference, said that the housing scheme they contemplated might be defined as the minimum of accommodation and convenience which every man and every family had a right to expect. Councillor Gray pointed out that in Glasgow out of a total of 156,000 occupied houses, 36,000 were houses of one apartment, and 70,000 houses of two apartments. Voluntary effort must be responsible for obtaining the land and capital necessary for the erection of cheap dwellings. Municipalities should have power to call upon owners of slum property to clear the land, and to insist that before being allowed to let property, owners should have a certificate of its suitability. Local authorities when securing land for parks or other public purposes should see that it was untrammelled with restrictions against building, so that a fair proportion of the land might be used for the purpose of building houses for the working classes. They should also have increased powers to enable them to compel owners of land in the immediate neighbourhood of the towns to sell their property at once, and not to withhold it until the prosperity of the towns had raised its value ten- or twenty-fold. Anderson at the same meeting asserted that the powers of corporations in regard to the demolition of slum property must be exercised cautiously and carefully. If there is not within a mile of these areas a sufficient number of houses of the same class and rental, the corporation should erect suitable tenements. Reasonable sanitary accommodation must be provided before demolition takes place. Municipalities should take every opportunity for securing whatever vacant ground there was for sale within reasonable distance. The Howard Association[2] draws attention to the fact that the scale of compensation which can now be demanded by owners of condemned areas is doubtless one of the main difficulties in the way of rehousing the poor upon such areas at a sufficiently low rental, thereby making the community pay for that which the owners of slum property are responsible. It should be made unprofitable to possess hopelessly insanitary property. The high rents charged for new dwellings tend to over-crowding the adjacent slums. Denmark grants State subsidies for land reclamation and forestry, and by the encouragement of many private undertakings, including co-operative dairy farming, the Danish agricultural classes have not only been retained upon the land, but an urban exodus into the country has been set up. The London County Council[3] decided to purchase, under the Housing Act, 31 acres of land at Norbury, in Surrey, on which to build cottages for artisans. It will afford accommodation when completed for about 5,800 persons. The plans provide for 551 single cottages of 3, 4, and 5 rooms each, and 211 double cottages containing tenements of 2 and 3 rooms. The rents proposed to be charged are, for single cottages, 3 rooms, 7s. to 9s. ; double cottages, 3 rooms, 6s. 6d. ; 2 rooms and small kitchen, 6s. ; 4 rooms, 9s. 6d. ; and 5 rooms, 11s. to 11s. 6d. The cost per acre of the site is about £600. The Report[4] of the Housing of the Working Classes Committee of the London County Council states that accommodation is now provided for 12,196 persons in its various buildings, which comprise 2,041 tenements, 305 cottages, and 324 cubicles. The gross total income for the year amounted to £44,226 12s. 3d., and of this 47·06 per cent. was required for outgoings during the year. The loss of rent by empties has been comparatively small. Fyfe[5] would make a distinction between "the working classes" and "the poor," and would designate as the latter all those earning on the average not more than 25s. per week, or households whose combined earnings per week do not exceed 30s. Two methods for adequately providing housing accommodation for such as these are available :—(1) To acquire land in the country and build cheap dwellings there, providing for them rapid transit at very low rates. (2) To acquire sites in the town and erect three- or four-storey blocks at distances convenient to their spheres of labour. The former is without doubt the ideal one, but since compulsion is out of the question the poor must be first educated as to the comparative advantages of country life, and tempted by financial considerations to leave the city life. The obstacles to this are the present railway and tramway fares, and it seems impossible to expect any solution of this problem until Parliament takes the whole question of these rates for the poorer classes into consideration. But country residence is not all. In town and country dwellings are built for poor people which are not, either in size or structural requirements, suitable as healthy houses. They are mainly defective in two considerations, viz., cubic capacity and effective through ventilation. The London County Council have erected houses of two apartments with a total of less than 1,400 cubic feet of free-air space. In Liverpool some of the most recently built two-apartment houses contain not more than 1,648 cubic feet. The minimum 2,150 cubic feet allowed by the London County Council is too limited for two apartments. The most recent houses of this description at Haghill contain considerably more than 3,000 cubic feet. Sykes[6] mentions that the problem of providing adequate house accommodation existed 300 years ago, and led to the introduction of laws prohibiting the building of houses for the lower classes within three miles of London and Westminster, and ordering newly-built houses to be demolished. At the present time the great expansion of commercial and professional activity is driving the resident population of the great towns from the centre to the periphery. This causes increased value of property, higher rentals, and consequently greater overcrowding in the cheaper quarters. Slums are manufactured by large houses, originally built for one family, being now let out in floors and tenements to numerous families. If the poorer classes are housed in such a manner, other classes must also suffer directly and indirectly. He quotes figures to show that a diminution in the size of dwellings tends to correspond with an increase in the mortality, particularly when accompanied by an increase in the number of persons per room, and that the progressive increase of mortality coincides with the progressive increase of the percentage of population living in dwellings of one room. Internal common staircases in compound dwelling-houses, by want of ventilation, are prejudicial to health, as they render the contained air common to all the dwellings opening upon them. It is a mistake to make large rooms for the poorer classes as it leads to overcrowding, the placing of more beds in the room, and the mixing of the sexes. In Glasgow, between the years 1871 and 1891, the persons living in one room fell from 30·4 to 18 per cent. of the total population, whereas those living in two and three-room dwellings rose exactly in the same proportion as those in the one room had fallen, namely, from 54·7 to 67·2 per cent. of the total population. In Liverpool[7] an association has been formed known as the "Liverpool Housing Association." Its object is to promote the erection, by the municipality, of healthy and comfortable homes for the people; to urge on the Legislature the necessity of simplifying the solution of the housing problem, particularly in respect of the taxation of land values, with option of purchase on basis of assessment. It is intended also to carry on a vigorous educational propaganda in order to rouse the citizens of Liverpool to a sense of the dangers and disgrace of insanitary dwellings, overcrowding, and slum life. Lantern lectures are given, the pictures including slums and slum dwellers, as well as those of model dwellings and villages. Symons[8] indicates the great need for action under the Housing of the Working Classes Act at Bath. In the Registrar-General's return for 1891, 4·11 per cent. of the population were considered to be living in overcrowded tenements. In 1891 there were in Bath over 3,000 persons living as families of three or more individuals in tenements of one or two rooms, and there were 39 persons living four, five, or six in a single room. He is of opinion that the least number of rooms for decent family life is three to each family, and as children of the two sexes grow up four rooms are necessary. A Provisional Order was obtained in 1896 for a street improvement, which necessitated the demolition of 17 dwellings occupied by the working classes. The Local Government Board approved the scheme in 1899, and in less than 12 months 16 houses had been completed, which it is proposed to let at sums varying from 5s. to 6s. 6d. per week. Altogether 40 dwellings will be erected, but owing to the rents they will be rented by the well-to do artisan class and not by the poorer classes who can only afford 2s. 6d. per week.

1 Public Health Engineer, Oct. 5, 1901. 2 Brit. Med. Jour., Nov. 24, 1900. 3 Brit. Med. Jour., Dec. 15, 1900. 4 Public Health Engineer, Nov. 16, 1901. 5 Sanitary Rec., Oct. 3, 1901. 6 Brit. Med. Jour., March 2 and 9, 1901. 7 Public Health Engineer, Nov. 16, 1901. 8 Brit. Med. Jour., July 20, 1901.

